# Feasibility of implementing a cellphone-based reminder/recall strategy to improve childhood routine immunization in a low-resource setting: a descriptive report

**DOI:** 10.1186/s12913-017-2639-8

**Published:** 2017-12-04

**Authors:** Victoria Bolanle Brown, O. Abimbola Oluwatosin

**Affiliations:** 1 School of Nursing, University College Hospital, Ibadan, Nigeria; 2 Department of Nursing, Faculty of Clinical Sciences, College of Medicine, University of Ibadan, Ibadan, Nigeria

**Keywords:** Childhood, Immunization, Reminder/recall, Acceptability, Adaptability, Effectiveness

## Abstract

**Background:**

Reminder/recall systems are effective ways to improve immunization rates, but their feasibility in primary health care (PHC) settings in Nigeria has not been adequately evaluated. In this study we describe the acceptability and adaptability of immunization reminder/recall system in an urban setting in southwest Nigeria.

**Methods:**

This is a descriptive report of a cluster randomized controlled trial. Four local government areas (LGAs) were randomly assigned into a cellphone reminder/recall intervention group or a usual care control group. Within each LGA, PHC centers were purposively selected to participate in the study. In each PHC center, mothers and their infants aged 0–3 months were enrolled into the two groups during the infants’ first immunization visit. Mothers (or other contact persons) in the intervention group received cellphone calls reminding them to take their child for scheduled immunizations. Follow-up of all the children lasted till the final scheduled immunization visit for each child. The intervention lasted for 13 months.

**Results:**

A total of 595 mothers/infants pairs (295 in the intervention group and 300 in the control group) participated in the study. Almost all mothers (*n* = 590, 99.2%) had access to their own cellphone or had access to a cellphone belonging to a significant other. Ninety-eight percent (*n* = 584) of all mothers were willing to receive immunization reminder/recall phone calls.

Eighty-seven percent (*n* = 2023) of all calls (*n* = 2324) for the reminder/recall intervention went through to the recipients and of these calls, 1948 (96.3%) were received. The mean cost of each call in US Dollars was about 5 cents. Immunization compliance rate (the receipt of required number of doses of routine vaccines at the appropriate age at recommended interval) was 79.2% among the children in intervention group and 46.4% in the control group (*p* < 0.001).

**Conclusion:**

Results demonstrate that cellphone reminder/recall interventions to improve routine childhood immunization are feasible in PHC settings in limited-resource settings with wide cellphone coverage, such as urban areas in Nigeria. Further research to test the potential for scale up in a variety of settings is recommended.

**Trial registration:**

PACTR201702002043415; Date of registration: 17 February 2017. (Retrospectively registered).

## Background

Preventable diseases are major causes of childhood morbidity and mortality world-wide [1]. Vaccine-preventable diseases (VPDs) constitute about a quarter of the eight million annual deaths among children under five children especially in low-income countries [2]. Immunization has been identified as one of the most effective public health interventions to reduce child morbidity and mortality [3]. However, poor compliance to immunization schedules and completion of recommended vaccinations limit the effectiveness of vaccination [4]. Globally, about 22 million infants are not fully immunized with routine vaccines and more than 1.5 million children under five years of age die from diseases that could be prevented by existing vaccines [5].

Fourteen percent of all incompletely vaccinated children globally live in Nigeria [6]. Compliance to and completion of recommended routine vaccines among children in Nigeria is sub-optimal with more than 3.2 million children aged 12 months old unimmunized, leading to outbreaks of VPDs across the country. Effective and novel strategies are therefore required to enable the country to meet the WHO recommended 95% level for the sustained control of VPDs and reduce under-five mortality.

Many studies have demonstrated the effectiveness of reminding families about scheduled immunizations and prompting clients who have missed a scheduled immunization appointment (recall) in improving vaccination rates [7–11]. Reminder and recall interventions have been found to be effective in various settings including family practices [12, 13], pediatric clinics [14, 15], and public health centers [16]. For example, the effectiveness of nurse-administered reminder interventions in improving immunization and other preventive visits in various practice settings have been demonstrated in studies in high-income countries [17, 18]. They have also been shown to be relatively easy to implement [19, 20]. However, the feasibility of cellphone-based reminder/recall interventions in PHC settings in low-resource contexts, such as Nigeria, has not been adequately evaluated. The current study fills this gap.

## Methods

### Aims

The aim of the study was to determine the feasibility of implementing a cellphone-based reminder/recall intervention designed to improve routine childhood immunization compliance (measured as the percentage of children correctly following immunization schedule) and coverage (measured as the percentage of fully-immunized infants) among infants in four local government areas (LGAs) in Ibadan, Oyo State, Nigeria.

In this study, the term ‘feasibility’ was used to capture the following elements: (1) Acceptability of childhood immunization reminder/recall system (mothers’ willingness to receive cellphone reminders and recalls); (2) Adaptability or practicability of implementing a childhood immunization reminder/recall intervention in PHC facilities without immunization registries or immunization information system; (3) Effectiveness of immunization reminder/recall system (the extent to which cellphone immunization reminder/recall intervention increases immunization compliance at PHC level in a low resource setting).

### Study setting

Ibadan is located in the south western part of Nigeria. It is the capital city of Oyo State and is located about 145 km north-east of Lagos, Nigeria’s commercial capital city. The projected 2015 population of Ibadan using 2006 population estimates and assuming a 3% annual population growth rate was 3.3 million [21]. There are 11 LGAs in Ibadan. The 2013 Nigeria Demographic and Health Survey showed that only 25.8% of children aged 12–23 months in Oyo State were fully immunized with recommended routine vaccines [22].

In 2015, Nigeria was ranked as the 9th highest country in cellphone usage out of 217 countries globally with about 83 subscriptions per 100 citizens [23]. In the same year, Oyo State had about 7.5 million mobile phone subscriptions [24]. In Nigeria, an individual can have multiple telecommunication subscriber identity module (SIM) cards with different cellphones. These reports have shed light on the potential feasibility of cellphone-based reminder/recall interventions in Nigeria.

### Design and sampling

We conducted a cluster randomized controlled trial targeting children aged 0–3 months at recruitment paired with their mothers in a larger study which aimed at assessing the effects of a community health nurse-led intervention on childhood immunization completion in the study communities [25]. The larger study was conducted between August 2012 and February 2014 while the trial occurred between August 2012 and September 2013.

Four randomly selected LGAs out of the 11 LGAs in Ibadan were allocated into a cellphone reminder/recall intervention and a control receiving usual care. One ward was randomly selected from each LGA and one PHC center with a large population of children who come for immunization was purposively selected from each ward. Each study group therefore had two PHC centers. Health care providers working in the PHC centers were not aware of the group allocations. The enrolment of eligible children into the two study groups was done during their first immunization visit, which is usually their first contact with the health center. Overall, 305 children were enrolled into the intervention group and 309 children into the control group.

Ethical approval for the study was obtained from the Oyo State Research Ethical Review Committee. All mothers provided signed informed consent prior to participating in the study.

### Intervention

After the enrolment of each eligible child into the study at the first immunization visit, mothers in the intervention group (or their primary contact) received one cellphone call reminder from the nurse/researcher two days before the child’s next immunization appointment and a second call a day before the appointment date. Recall phone calls were made for missed appointments. If a child was not brought on the scheduled immunization day, the nurse/researcher automatically re-scheduled the child for the next immunization day. The pattern of recall cellphone calls was similar to that for the reminder phone calls. Follow-up of the children in the intervention and control groups lasted till the last scheduled immunization visit for each child. Phone calls were made between 9.00 am and 8.00 pm.

### Instruments

Data were collected using three questionnaires and one checklist. The first questionnaire was used to gather information on the socio-demographic characteristics of children and their parents, parents’ phone usage and mothers’ willingness to receive immunization reminder/recall phone calls. The second questionnaire recorded the children’s immunization data. The third questionnaire, which was adapted from an American Immunization Registry Association guidebook [26], documented reminder/recall activities for each child in the intervention group. The checklist was used for weekly tracking and follow-up of children due for immunization, and also for rescheduling missed immunization appointments.

### Data analysis

The socio-demographic characteristics of the two groups were compared using the chi-square test or Fishers exact test as applicable. The primary outcomes for this study were the proportion of mothers who accepted to receive reminder calls, the proportion of calls that were made and received, and the proportion of children who complied with the immunization schedules. Statistical significance was set at *p* < 0.05. All statistical analyses were performed using SPSS version 22 software (IBM Corporation, Armonk, NY).

## Results

**Fig. 1:**
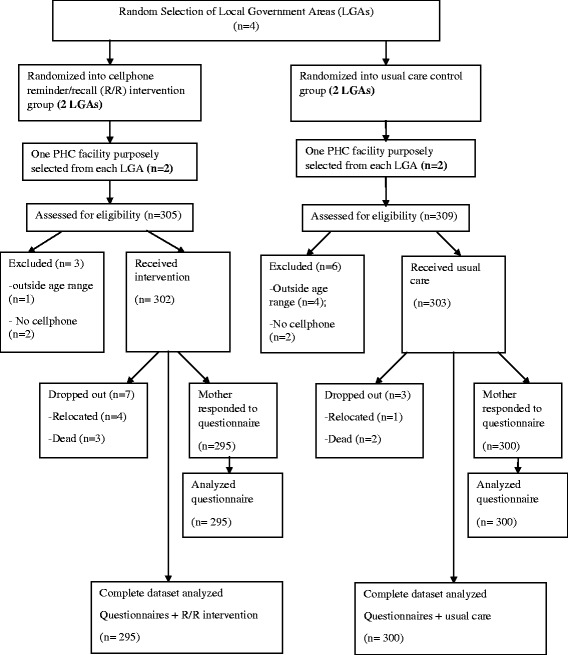
Study flowchart

Overall, 614 eligible children aged 0–3 months were enrolled at the commencement of study into the intervention and control groups. Data from 19 (3%) children were excluded from analysis resulting in an analytical sample of 595 children (295 in the intervention group and 300 in the control group). The study participants’ flow chart is presented in Fig. 1.

The socio-demographic characteristics of the children in the two groups are presented in Table 1. The mean age of the children at enrolment was 14.6 days (SD 14.5) in the intervention group and 18.3 days (SD 16.4) in the control group. Significant differences between the groups were noted in the mean age (in days) at first immunization visits (*p* < 0.05). Maternal age did not differ significantly between the two groups (*p* = 0.88).

**Table 1:** Comparison of socio-demographic characteristics of participating children between study groups

Variables	Study groups					
	Reminder/recall Intervention		Control (usual care)		χ^2^	*p* value
	Frequency	%	Frequency	%		
Gender					4.751	0.033
Male	130	44.1	159	53		
Female	165	55.9	141	47		
Family Type					3.276	0.227^a^
Monogamy	280	94.9	276	92		
Polygamy	11	3.7	21	7.0		
Single mother	4	1.4	3	1.0		
Birth Order					1.374	0.503
1	98	33.2	97	32.3		
2–3	157	53.2	152	50.7		
≥ 4	40	13.6	51	17.0		
Family Religion					13.129	<0.001
Christianity	212	71.9	173	57.7		
Islam	83	28.1	127	42.3		
Maternal Education					9.877	0.007
Below secondary	19	6.5	39	13		
Secondary	165	55.9	138	46		
Post-secondary	111	37.6	123	41		
Mother’s Employment Status					8.761	0.067
Unemployed	37	12.5	22	7.3		
Petty trading	130	44.1	163	54.3		
Artisan	82	27.8	73	24.3		
Civil servant	39	13.2	33	11		
Others	7	2.4	9	3.0		
Place of Delivery					9.456	0.024
Public health facility	72	24.4	63	21		
Private health facility	100	38.9	138	46		
Mission/TBAs	104	35.3	86	28.7		
Home	19	6.4	13	4.3		

^a^Fisher’s Exact Test

### Acceptability of immunization reminder/recall system

Almost all, 590 (99.2%) of the mothers of the children had access to a cellphone with a valid number (either their own or belonging to someone else). These mothers provided one to five valid cellphone numbers where they could be reached. Ninety-eight percent (*n* = 584) of mothers agreed to receive immunization reminder/recall phone calls (Table 2).

**Table 2:** Mothers’ Willingness to Receive Immunization Reminder/Recall

Variable	Response			
	Yes		No	
	Frequency	%	Frequency	%
Are you willing to be receiving reminder/recall about your child immunization?	584	98.2	11	1.8
Would you be willing to record your cellphone number at the immunization clinic to receive phone calls about your child’s immunization?	570	95.8	25	4.2
Would you be willing to be reminded of your child’s immunizations before the appointment day	556	93.4	39	6.6

### Adaptability of immunization reminder/recall system

Of the total 1162 cellphone calls made, 974 (83.8%) were reminder calls. Of the 974 reminder calls, only 41 (4.2%) were not received by the recipients. Similarly, only six (3.2%) of recall calls were not received. Eighty-five percent (*n* = 983) of all calls went through on the first day of each session of intervention. On the second day, a total of 1049 (90.3%) of all the 1162 sessions of calls went through (Table 3).

**Table 3:** Cellphone Reminder/Recall Activities in the Study

Cellphone reminder/recall Activities	Yes		No	
	Frequency	%	Frequency	%
Call went through the 1st day of intervention (*n* = 1162)	983	84.6	179	15.4
Call answered the 1st day of intervention (*n* = 983)	695	98.2	18	1.8
Call went through the 2nd day of intervention *n* = 1162	1049	90.3	113	9.7
Call answered the 2nd day of intervention (*n* = 1049)	1018	97.1	31	2.9

As shown in Table 4, mothers were the most frequent recipients of phone calls for the reminder/recall intervention. A maximum of five attempts were made if there was no answer or a busy signal. The mean duration for the calls was 29 s per session on the first day and 23 s per session on the second day of the intervention. The mean cost in US Dollars was about 5 cents per session on the first day and about 4 cents per session on the second day of the intervention.

**Table 4:** Recipients of Cellphone Calls

Recipients of cellphone calls	1st day (*n* = 983)		2nd day (*n* = 1049)	
	Frequency	%	Frequency	%
Child’s mother	708	72.1	702	67
Child’s father	249	25.4	316	30
Child’s older sibling	1	0.1	0	0
Maternal grandparent	12	1.1	15	1.4
Paternal grandparent	7	0.7	9	0.9
Others (Aunt, friend, neighbor)	6	0.6	7	0.7
Total	983	100	1049	100

### Effectiveness of immunization reminder/recall system

**Fig. 2:**
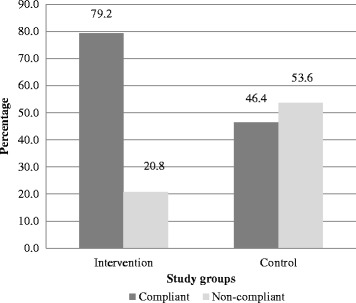
Immunization compliance rates between intervention and control groups

The main trial outcome has previously been reported elsewhere [25]. Using DPT 3 coverage (which is a key indicator for assessing the effectiveness of childhood immunization services) [27], compliance rate was 79.2% for the intervention group and 46.4% for the control group (*p* < 0.001) (Fig. 2).

## Discussion

The results from this study demonstrate the feasibility of a cellphone-based childhood immunization reminder/recall system in a low-resource setting, such as Nigeria. Almost all mothers had access to a cellphone and were willing to record their phone numbers in the clinic and to receive reminder/recall phone calls. Previous studies have shown mothers’ preference for and acceptability of cellphone calls for immunization reminder/recall [28–30]. Importantly, results demonstrated the effectiveness of the reminder/recall intervention with over three-quarters of the children in the intervention group complying to the recommended immunization schedule compared to about half of the children in the control group.

Majority of the calls made were for reminders about scheduled appointments rather than follow-ups for missed appointments. These results suggest that gentle reminders by health workers can boost immunization compliance. Furthermore, the fear that the strategy may be costly appeared to be largely unfounded as calls were relatively inexpensive and there was often no need to make a recall phone call for missed appointments for majority of the participants.

Although mothers received most calls, the reminder messages seemed to have been conveyed by other contact persons listed during enrollment. This finding suggests the importance of the existing family support system in Nigeria [31–33]. This system needs to be strengthened because of the possible contribution of social support to positive child health outcome and health care outcomes in general [34, 35].

We were able to reach mothers and other contacts through calls primarily made during working/business hours. However, further research exploring the use of text messages may be warranted to test feasibility in situations where parents are working in settings where phone calls are not allowed during working hours. Also consent may be taken from such parents regarding the time of the day that they are free to receive immunization reminder/recall phone calls.

We found that the cost of cellphone calls for reminder/recall intervention was relatively inexpensive and that making the calls was not time consuming. Thus, this strategy can be implemented in low-resource settings. Further, record clerks who normally schedule clients’ appointments can be guided or trained to make reminder/recall phone calls to reduce time demands on nurses and other immunization providers given the shortage of health workforce in many low and middle income countries [36–38]. However, other administrative support infrastructure like comprehensive immunization registers, clients’ phone numbers, electricity to charge cellphones and phone call log books should be available.

Overall, the study revealed the effectiveness of cellphone immunization reminder/recall intervention in increasing immunization completion rates and adds to the body of evidence demonstrating the effectiveness of the intervention across a range of settings where the availability of technology to provide reminders exists [39].

### Strengths and limitations of the study

Study findings should be interpreted in light of several limitations. First, the study was based on a sample of children and their mothers recruited during the time of first immunization rather than a random community sample. The study therefore possible targeted those already predisposed to complete their vaccination. However, previous studies in Ibadan, Nigeria [40–42] have found that many children who commence routine immunization do not complete the recommended vaccines. Second, the study was conducted in an urban setting and results may not be generalizable to rural and peri-urban settings. Third, only the costs of cellphone calls were captured in the study. Other costs, such as staff time and expenses for equipment and supplies were not captured. Thus, a comprehensive assessment of the cost of the intervention was not possible. Despite these limitations, this study demonstrates the acceptability, adaptability and effectiveness of a cellphone-based routine childhood immunization reminder/recall intervention in a low-resource setting in Nigeria.

## Conclusion

This study’s results demonstrate that the use of client reminder/recall systems can provide community health nurses and other public health professionals with real-life experience of community-based practice that can improve the health of the populations they serve. The use of electronic communication technology in public health interventions can improve clients’ adherence and compliance to guidelines related to their treatment, health promotion and diseases prevention. In addition, results suggests that simple, paper-based immunization information systems can be effective if well-conceived for data collection and use in low-resource settings. Further research to test the potential for scale up in a variety of settings is recommended.

## Abbreviations

DPT: Diphtheria, pertussis, and tetanus
LGA: Local government area
PHC: Primary health care
VPDs: Vaccine-preventable diseases
WHO: World Health Organization
